# Tumor therapeutics in the era of “RECIST”: past, current insights, and future prospects

**DOI:** 10.3389/or.2024.1435922

**Published:** 2024-10-18

**Authors:** Zhilong Xu, Gening Jiang, Jie Dai

**Affiliations:** Department of Thoracic Surgery, Shanghai Pulmonary Hospital, Tongji University School of Medicine, Shanghai, China

**Keywords:** response evaluation criteria in solid tumors (RECIST), immune checkpoint inhibitors (ICIs), liquid biopsy, artificial intelligence (AI), tumor imaging

## Abstract

In recent years, advancements in medical treatment and imaging technologies have revolutionized the assessment of tumor response. However, the Response Evaluation Criteria in Solid Tumors (RECIST) has long been established as the gold standard for evaluating tumor treatment. As treatment modalities evolve, the need for continuous refinement and adaptation of RECIST becomes increasingly apparent. This review explores the historical evolution, current applications, limitations, and future directions of RECIST. It discusses the challenges of distinguishing true progression from pseudo-progression in ICIs (immune checkpoint inhibitors), the integration of advanced imaging tools, and the necessity for RECIST criteria tailored to specific therapies like neoadjuvant treatments. The review highlights the ongoing efforts to enhance RECIST’s accuracy and reliability in clinical decision-making and the potential for developing new standards to better evaluate treatment efficacy in the rapidly evolving landscape of oncology.

## 1 Introduction

The continuous advancement in tumor therapeutic technologies provides us with more options, including chemotherapy, radiotherapy, immune checkpoint inhibitors (ICIs), targeted therapy and so on. Although the ultimate goal of cancer treatment is “cure,” it remains a challenging task for the majority of tumors at present (1). Initial prognostic assessment stems from the summarization of clinical experiences. A clear conclusion is that patients with a significant reduction in tumor size after treatment typically exhibit better prognosis (2). Assessment of tumor treatment response is crucial for clinical trials and the selection of cancer treatment regimens, which is the basis of clinical decision-making. The assessment of tumor burden has become a crucial component of most clinical trials. In 1979, the World Health Organization (WHO) proposed RECIST (response evaluation criteria in solid tumors) to provide a consistent framework for evaluation. The implementation of standardized definitions ensures uniform assessment of responses across institutions in multicenter trials and facilitates comparison of treatment responses across different trials. RECIST is widely used to evaluate various tumor types and studies. Although RECIST was initially developed to assess treatment activity in early phase II trials with tumor response as the primary endpoint, it has since been extended in practice to encompass the entire range from early phase I trials to confirmatory phase III trials. Additionally, RECIST criteria are included in endpoint definitions such as response rate and progression-free survival.

However, since the advent of ICIs, a different pattern of post-treatment response assessment has emerged. This typically presents as atypical response patterns, also referred to as immune-related clinical response patterns (3). With further research, additional response patterns have been identified, such as pseudo-progression (PsPD), delayed response, and hyper-progressive disease (HPD) (4, 5). The presence of these outcomes presents increasingly significant challenges for clinical practitioners in assessing the clinical efficacy of ICIs and making clinical decisions, particularly when imaging indicates initial or new lesions leading to increased tumor burden, potentially resulting in misjudgment of patient outcomes and lead to unnecessary stop. Therefore, how to assess the efficacy of ICIs or targeted therapy post-treatment, as well as identifying potential beneficiaries early on, will directly impact the formulation and adjustment of clinical treatment decisions.

In this review, we have reviewed various RECIST criteria that have emerged to date, along with their limitations (some of which are still in use). We discussed concepts of new methods and biomarkers, as well as the possibility of incorporating them into the RECIST framework.

### 1.1 The evolving RECIST standards adapt to the continuously progressing landscape of tumor therapeutics

**FIGURE 1:**
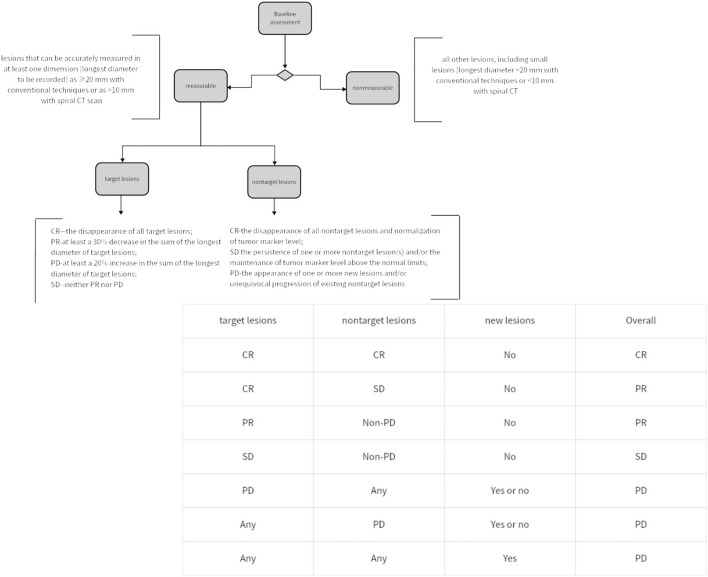
Simplified assessment workflow of RECIST criteria and methodology for overall status evaluation.

In 1979, WHO pioneered a clinically experienced-based assessment standard for evaluating the impact of cancer treatment. This standard, for the first time, defined disease response as a 50% objective reduction in lesion size, while disease progression was defined as an increase in lesion size exceeding 25% (6). In 2000, the European Organisation for Research and Treatment of Cancer proposed the Response Evaluation Criteria in Solid Tumors for assessing solid tumor response. The RECIST criteria proposed concepts such as Complete Response, disappearance of all target lesions (CR), Progressive Disease, 20% increase (PD), Partial Response, 30% decrease (PR) and Stable Disease (SD), Neither PR nor PD criteria met). Within the RECIST criteria, it is specified that a maximum of five lesions per organ and a maximum of ten lesions in total can be selected. Additionally, it is proposed to categorize tumor lesions as measurable (those that can be accurately measured in at least one dimension [the longest diameter should be recorded], using conventional techniques ≥20 mm, or using spiral CT scan ≥10 mm) and non-measurable (all other lesions, including small lesions [the longest diameter <20 mm using conventional techniques or <10 mm measured with spiral CT scan] and truly non-measurable lesions) (7). The specific criteria of RECIST can be found in Figure 1. Since then, RECIST has served as a standardized method for assessing tumor response in advanced solid cancers, and it has been validated across various types of tumors for its predictive value in overall survival (OS). The proposal of RECIST standard leads to comparable results by determining the new cut-off value of reaction and disease progress, which indicates that we can compare the results of different clinical trials (8). However, in clinical practice, it has been observed that RECIST assessment performs well in evaluating tumor shrinkage efficacy, but it is less effective in assessing measures beyond tumor reduction.

Compared with RECIST, RECIST 1.1 reduces the total number of lesions to be evaluated from 10 to 5, and the maximum lesions of each organ from 5 to 2 (9). In addition, RECIST 1.1 also increases the evaluation of lymph node lesions. Most importantly, the definition of PD in RECIST 1.1 is increased by 20% or more from the original target organ lesions, and the requirement that the absolute value of 5 mm should be increased is also added. Additionally, guidance is provided for “unequivocal progression” of non-measurable/non-target lesions. In the initial standard, bone metastases were considered unmeasurable due to the lack of sensitivity in detecting bone marrow infiltration with the technology available at the time. However, in RECIST 1.1, bone metastases ≥10 mm in soft tissue are identified as measurable target lesions, attributes to advancements in imaging technology.

As more immunotherapies are being applied in clinical practice, especially ICIs, it has become increasingly evident that the imaging manifestations of patients receiving ICIs differ from those undergoing traditional cytotoxic treatments, exhibiting an atypical pattern. One of the atypical features of ICIs response-related tumor burden, as compared to cytotoxic therapy, is the presence of delayed responses (10). ICIs takes longer to become effective, hence the longer response delay time. SD is also considered an indicator of treatment efficacy in immunotherapies assessment after 2–3 cycles treatment. Additionally, during immunotherapies, there may be lesion enlargement and the development of new lesions (11). According to previous understanding, this disease would be classified as PD. However, with research into the mechanisms related to lesion enlargement and development, possible reasons could be that treatment-induced immune responses against tumor cells may lead to the influx of inflammatory cells into the tumor, resulting in a transient increase in tumor size, which may be confused with disease progression (hence termed “pseudo-progression”) (12). It is worth noting that a few immune-related adverse events (irAEs) caused by ICIs may be confused with PD. For example, the treatment of metastatic melanoma with nivolumab may induce pulmonary sarcoid‐like granulomatosis (13).

### 1.2 In the era of immune checkpoint inhibitors

RECIST 1.1 has significant limitations in assessing the efficacy of ICIs. To address the challenges in evaluating tumor response under ICIs, Wolchok et al. first proposed the modified “Immune-Related Response Criteria” (irRC) in 2009, based on the WHO standards (14). In 2014, it further evolved into irRECIST at the European Society for Medical Oncology (ESMO) congress (14). irRECIST considers only a significant increase in tumor lesions (irRC ≥25%; irRECIST ≥20%) as indicative of tumor progression (iPD = “immune-related progressive disease”). Additionally, non-target organ lesions should also be considered when evaluating PD. Cases assessed as PD should be reassessed at least 4 weeks later to avoid interference from pseudo-progression (15, 16). On 2 June 2016, a meeting was held in Chicago, Illinois, United States, where based on previous experience, the response criteria following ICIs were standardized, known as iRECIST (17). iRECIST is developed based on RECIST 1.1, with advancements including the introduction of a new concept, unconfirmed progressive disease (iUPD), to refer to the status of patients who have not been diagnosed with iCPD (immune-*confirmed PD*). Multiple iUPDs are allowed, and they can persist in subsequent assessments until they convert to iCPD or to iCR (immune-complete response), iPR (immune- partial response), or iSD (immune stable disease). The concept of iUPD helps further understanding and better description of atypical responses following ICIs, including pseudo-progression and delayed responses. Regarding new lesions, the change made by iRECIST is that newly identified lesions meeting the criteria for iUPD should continue the previous treatment under the premise of clinical stability in patients until the next assessment (≥4 weeks later). A survival analysis study by Tazdait focusing on late-stage Non-small cell lung cancer population treated with ICIs demonstrated that iRECIST, compared to RECIST 1.1, better identifies survival benefits from immune checkpoint inhibitors for some (13/120) previously classified as “PD” patients (18). Another study by Rebuzzi reported a case of advanced renal cancer patient who experienced PD status after treatment with nivolumab, yet the patient still derived clinical benefits from subsequent ICIs and exhibited delayed radiation response following initial progression (19). However, some studies have also found that in the assessment of ICIs, iRECIST has an advantage only in certain specific treatment categories, such as subgroups of anti-CTLA-4 antibody treatment, while in other treatment types, there is no significant difference between the two (20). Similar conclusions were drawn in another meta-analysis (21). To address the discrepancies between the two, more samples will be needed in future studies to arrive at more convincing conclusions.

With the widespread development of new immunotherapeutic agents for treating various types of cancers, standards are continuously revised and matured to robustly assess efficacy and benefits. The 2018 Immune-Related Response Evaluation Criteria in Solid Tumors (imRECIST) aims to better capture immune therapy responses (22). imRECIST, like iRECIST, does not consider the PD status as a response event endpoint in survival analysis until it is confirmed. Both iRECIST and imRECIST are generally classified as immune therapy evaluation criteria, emphasizing that new lesions do not always represent PD compared to conventional criteria. Additionally, PD does not always imply treatment cessation (besides PD, there may still be potential benefits from treatment). Although the introduction of immune therapy-related criteria allows for the existence of pseudo-progression, there is still no definitive method for identifying pseudo-progression. Therefore, the current assessment of tumor response under immune therapy urgently requires distinguishing between the concept of pseudo-progression and actual progression.

The advent of intratumoral (IT) ICIs has rendered iRECIST and imRECIST ineffective in assessing local interventions, as they are entirely based on RECIST 1.1, which does not permit separate evaluation of injected and non-injected lesions. Consequently, in 2020, itRECIST (*intratumoral RECIST*) was introduced as a guideline for data collection and response assessment in clinical trials of IT ICIs (23). Compared to other versions, itRECIST specifically divides the collection of baseline lesion data into four parts: target-injected (T-I), target-non-injected (T-NI), non-target-injected (NT-I), and non-target-non-injected (NT-NI). The remaining assessment methods are similar to RECIST 1.1. However, it is worth noting that itRECIST allows for ultrasonographic measurements of lesions beneath the skin, which were not previously mentioned in existing guidelines, and extends the interval for confirmatory reassessment from the 4- to 8-week timeframe of RECIST to allow for reassessment between 4 and 12 weeks.

### 1.3 Assessing tumor prognosis with PET-CT—PERCIST

The widespread adoption of advanced imaging technologies has increased the possibilities for multidimensional assessment of tumor prognosis, such as metabolic imaging. However, standardized and unified criteria are also required in this regard. In addition to tumor volume, the metabolic activity of tumors through functional imaging techniques (e.g., positron emission tomography - PET) has been found to be highly predictive of responses in lung cancer and melanoma (24, 25). For example, there seems to be a close relationship between the uptake of 18F-FDG and the quantity of cancer cells. As far back as 1993, 18F-FDG PET was discovered to be predictive of tumor response in breast cancer (26). 18F-FDG PET has also been found to be associated with the prognosis of tumors such as esophageal, pulmonary, head and neck, and lymphoma. Specifically, parameters such as standardized uptake value (SUV), influx rate of 18F-FDG (influx constant Ki), and phosphorylation rate of FDG-6 phosphate (k3) have shown to be lower in patients with a favorable prognosis compared to those with a poor prognosis, and this change occurs earlier than changes in the size of the tumor itself (27). With the increasing utilization of PET, there is a growing demand among researchers for standardized and uniform PET treatment response metrics. Consequently, the Positron Emission Tomography Response Criteria in Solid Tumors (PERCIST 1.0) was proposed in 2009 (28). Meta-analysis indicates that PERCIST is more suitable as an independent prognostic factor for survival and is better suited for evaluating tumor response to anticancer therapy compared to RECIST (29). However, PERCIST also faces some unresolved issues. After ICIs, inflammation effects arising from immune system activation and subsequent infiltration of lymphocytes into tumors may impact the assessment of tumor response using 18F-FDG-PET, which could be perceived as pseudo-progression (30, 31). However, 18F-FDG also has some problems that have not been overcome. For example, 18F-FDG accumulates not only in tumor cells, but also in some inflammatory sites with high metabolic activity, which may lead to false positive results, such as in the case of inflammatory diseases or infections; Secondly, the uptake of 18F-FDG by some types of tumors (prostate cancer) is low or not obvious. This may lead to false negative results. This should be considered more in the future PERCIST standard.

In addition to 18F-FDG, 67Ga is gradually being incorporated into the assessment of treatment efficacy due to its excellent imaging capabilities. Tumor uptake of 67Ga is considered one of the ways to distinguish between “hot” and “cold” tumors (32, 33). It is often believed that “hot” tumors, which exhibit higher levels of inflammatory infiltration, have a better response rate to ICIs compared to “cold” tumors (34, 35). 67Ga has been found to have prognostic value in lymphomas, lung tumors, breast tumors, malignant melanoma, testicular tumors, and brain tumors (36, 37). However, it is worth noting that some studies suggest the prognostic significance of negative 67Ga uptake often outweighs the significance of positive uptake. This is because the characteristics of the radiotracer itself may seek out tumor properties. It is worth noting that there are many other radioligands used for detecting treatment responses in cancer, such as 18F-fluoroestradiol (FES) in hormone-dependent breast cancer and 18F- or 68Ga-prostate-specific membrane antigen ligands in prostate cancer (38–40).

### 1.4 Another pathway for RECIST—liquid biopsy

**FIGURE 2:**
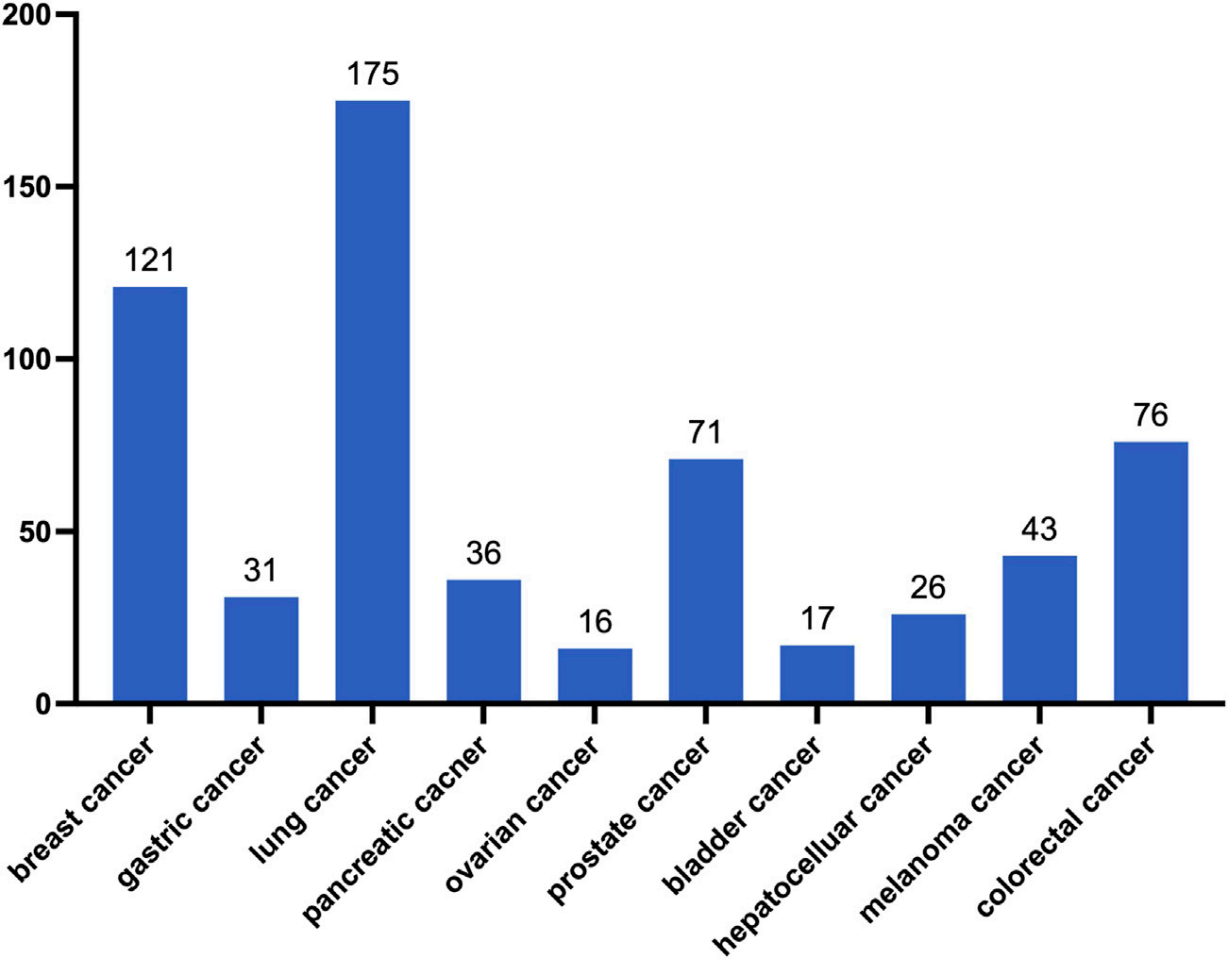
Number of CTC-related studies registered on the National Institutes of Health website (as of March 2024).

Liquid biopsy (LB) analyzes tumor cells or products (CTCs, cfRNA, ctDNA, TEPs, etc.) from tumors in the bloodstream or fluids (41, 42). In breast cancer, lung cancer, prostate cancer and colorectal cancer, circulating tumor cells (CTC) show good early prediction ability (43–50). Therefore, there is considerable research focusing on the relationship between CTCs and the assessment of tumor treatment outcomes. A search on the National Institutes of Health’s official website (http://clinicaltrials.gov/ct2/home) using the keyword “circulating tumor cells” (as of March 2024) revealed 683 ongoing clinical studies (Figure 2). Circulating tumor DNA (ctDNA) is free DNA from cancer cells in the bloodstream, linked to tumor prognosis. Higher ctDNA mutation burden correlates with survival in non-small cell lung cancer (51, 52). Additionally, ctDNA can be used for monitoring disease recurrence. For example, Newman reported two stage IB patients treated with surgery and SBRT who had undetectable ctDNA and long-term survival. A stage IIIB patient, despite a complete response on imaging after chemotherapy, had rising ctDNA and disease recurrence (53). Whats’ more, a range of biomarkers, such as peripheral blood lactate dehydrogenase and interleukin-8 levels, as well as the neutrophil-to-lymphocyte ratio, have been shown to aid in evaluating immune response patterns (54).

With the widespread adoption of liquid biopsy techniques, there is an urgent need for standardized detection methods and precise definitions for assessing oncological ctDNA responses and/or progression. Most studies have found that a reduction in ctDNA during treatment corresponds to a better prognosis, but there is no consistent standard for quantifying this reduction. Some researchers suggest that a relative decrease to a low value is sufficient, while others advocate for undetectable levels as the response criterion. Similar issues arise in defining criteria for ctDNA increase and ctDNA progression. In this context, Anders K M Jakobsen proposed ctDNA-RECIST as an alternative to RECIST, offering a framework for assessing ctDNA responses in oncological contexts (55). Firstly, Anders posited that the criterion for a significant decrease in ctDNA should be lower than the previous measurement, with no overlap in the confidence intervals between the two measurements. Subsequently, Gouda et al. proposed the plan for the Liquid Biopsy Response Evaluation Criteria in Solid Tumors (LB-RECIST) in March 2024 (56). The authors propose that the development of LB-RECIST should parallel RECIST 1.1 and outline five key questions that need to be addressed urgently. First, “what” - while plasma is currently the main source for ctDNA detection, further evaluation of plasma versus other sources of ctDNA, especially in certain specific diseases such as gallbladder cancer, is warranted. Second, “who” - as mentioned earlier, ctDNA was initially focused on assessing advanced diseases, but with increasing evidence of its feasibility for early assessment, different standards should be proposed to limit its scope when dealing with different study subjects. Third, “when” - there should be a unified standard for the timing of ctDNA assessment. Currently, the timing of ctDNA collection in research mostly depends on the subjective decisions of researchers. In the future, the impact of different time points of ctDNA assessment on evaluation outcomes in certain diseases should be addressed, in order to select the most appropriate timing and establish a paradigm. Fourth, “why” - the impact of ctDNA versus radiological assessment on long-term clinical outcomes and their respective applicability needs further research and clarification in the future. Fifth, “how” - the technology and platforms for detecting ctDNA are constantly evolving, and appropriate technologies and platforms should be selected for different diseases. In addition, the authors have designed a potential validation scheme: patients will be enrolled into either the ctDNA-guided arm or the standard control arm. Patients in the ctDNA-guided arm will undergo evaluation with a drug associated with shorter progression-free survival (PFS) after 2 weeks of treatment initiation. Patients with RECIST PR in ctDNA will continue the initial treatment, while those with RECIST SD or RECIST PD will switch to a second drug for treatment. Patients in the standard arm will receive the same initial treatment and will be randomized to either continue the same treatment or switch treatment after 2 weeks. All patients will undergo evaluation via radiological examination at 8 weeks.

RECIST standards have evolved over decades, LB-RECIST is currently in its early stages. However, it is anticipated that with the continuous advancement of new technologies and research, the significance of LB-RECIST will become increasingly prominent. The traditional RECIST standard relies on imaging data, while liquid biopsy relies on molecular data in blood samples. This difference in data sources may bring difficulties in data integration. Finally, how to combine the dynamic changes of ctDNA with imaging evaluation to form a comprehensive evaluation standard is an important issue in current research. In the future, we need to explore this aspect in order to optimize the combination of liquid biopsy and RECIST and improve the accuracy and reliability of cancer treatment effect evaluation.

### 1.5 Artificial intelligence is gradually becoming involved in RECIST

Artificial intelligence (AI) has the capability to transform digital medical images into high-dimensional quantitative data, which can be utilized to evaluate tumor biological characteristics, thus paving the way for a new avenue in medical research. AI is often employed to assist clinical practitioners in making prognostic decisions for patients. A study involving 43 patients with liver metastases (both previous and current scans) demonstrated that the accuracy of AI-assisted readings improved by 34.5% compared to judgments made independently by conventional researchers. The impact of AI on the results primarily stems from its superior quantification of liver lesion volumes compared to the control group (57). Another study developed an interactive LiTS (Liver Tumor Segmentation) method based on deep learning (DL) for accurately measuring the boundaries of liver tumors to estimate the size of lesions in target organs (58). In a study on malignant pleural mesothelioma, researchers also conducted fully automated volume measurements based on DL and generated volume reports independently for the first time without human intervention, producing prognostic information almost identical to that generated by human readers based on the modified RECIST standard (59). In the process of AI technology development, significant optimization has been achieved in image generation, reconstruction, and measurement of relevant dimensional data, enabling strict adherence to the RECIST standard. However, with such precise metrics, it raises the question of whether RECIST should be further updated to address the ambiguities that were previously considered in light of technological accuracy. This is one of the future considerations.

Another advantage of AI lies in its ability to generate new paradigms by identifying structural patterns in data that are not readily apparent to humans. CT texture analysis (CTTA) reflects local spatial variations in image brightness and serves as a tool for analyzing CT heterogeneity. Clinical studies have shown that CTTA can provide independent prognostic indicators for patients with non-small cell lung cancer, esophageal cancer, colorectal cancer, head and neck cancer, and renal cancer (60–64). However, using CTTA as a measure of tumor characterization, prognosis, and response assessment requires a higher level of sophistication, made possible with the assistance of AI. In an experimental study involving patients with metastatic melanoma treated with the PD-1 inhibitor nivolumab, radiomic texture features extracted from CT scans using machine learning methods were identified as important prognostic factors for survival in patients receiving nivolumab monoclonal antibody treatment (65).

What is even more exciting is that the introduction of AI promises a highly promising future for more early response imaging markers in predicting prognosis, treatment response, and tumor phenotypes. In simple terms, it involves extracting patterns (imaging biomarkers) from a set of imaging data and then making predictions based on statistical data. Kathryn C. Arbour employed deep learning methods in a study in 2020 to evaluate the treatment outcomes of non-small cell lung cancer patients treated with PD-L1 therapy (66). The model achieved an accuracy rate of 90% for assessing progression in 92 cases and correctly predicting the RECIST progression date in 79% of cases. However, a major limitation of this model is its exclusion of cases of pseudo-progression, making it unsuitable for evaluating pseudo-progression. In a study on ICIs response prediction in stage IV melanoma patients, machine learning achieved an Area Under the Curve (AUC) of 0.85 (67). Zhu et al.’s study found that deep learning methods could be utilized to predict tumor response to chemotherapy in patients with colorectal liver metastases (68). The integration of AI with RECIST holds the potential to address inherent delays in the RECIST standard itself, thereby opening up new possibilities for early treatment response assessment biomarkers. Additionally, the utilization of such deep learning or machine learning models can significantly augment the frequency of RECIST standard assessment queues, suggesting the potential utilization of datasets beyond clinical trial settings (real-world evidence - RWE) for predicting patient prognosis in the future (66, 69). The widespread utilization of artificial intelligence is paramount for clinical treatment decision management and the proposal of novel treatment approaches.

Currently, the field of AI is still in its nascent stages, with limited external validation evidence available for existing models, and their performance has yet to be validated in real-world settings. There is much to learn regarding the potential applications of deep learning and machine learning. Additionally, a crucial aspect of projects involving AI integration is the standardization of radiological processes, such as preprocessing and modeling, to ensure uniform data extraction, guided by international consensus guidelines and/or cumulative evidence. Whats’more, implementing artificial intelligence in clinical environment also faces potential ethical and practical challenges. First of all, the decision-making process of AI model is often “black box” and lacks transparency, which may make it difficult for clinicians to understand and explain the prediction results of the model, thus affecting patients’ informed consent.

### 1.6 The future of RECIST

Although the RECIST standard is constantly developing with the addition of new contents and standards, the improved RECIST1.1 standard is widely used as the gold standard for evaluating therapeutic activities. The original RECIST standard creatively introduced many concepts, and laid the foundation for the basic framework of today’s tumor treatment efficacy evaluation experiment. Different versions of RECIST have varying scopes of applicability and definitions (Table 1). With the continuous verification of its feasibility in practice, RECIST1.1 is regarded as a relatively recognized evaluation standard in most clinical experiments at present.

**TABLE 1:** Summary comparison of various RECIST criteria.

	RECIST	RECIST1.1	irRECIST	iRECIST	imRECIST	itRCEIST	PERCIST
Applicability	Solid tumor	Solid tumor	immunotherapy	immunotherapy	immunotherapy	Intratumoral immunity, tumor ablation	Metabolic imaging
Number of lesions evaluated	Uni-dimensional ≥10 mm, 10 lesions, 5/organ	Uni-dimensional ≥10 mm, 5 lesions, 2/organ	Uni-dimensional, ≥10 mm, 5 lesions, 2/organ	Uni-dimensional, ≥10 mm, 5 lesions, 2/organ	Uni-dimensional, ≥10 mm, 5 lesions, 2/organ	Uni-dimensional, ≥10 mm, 10 lesions (5 injected, 5 not injected)	5 lesions, 2/organ
Confirmation of PD	20% increase	Target organ lesions increased by 20%, while meeting the absolute increase of 5 mm	Re-evaluate after 4–12 weeks	Re-evaluate after 4–8 weeks	Re-evaluate at least 4 weeks	Re-evaluate after 4–12 weeks	18F-FDG SUL increased by > 30%, and the absolute value was > 0.8 SUL unit or the absorption degree of 18F-FDG tumor increased obviously (TLG volume was 75%, but SUL did not decrease
New lesions	PD	PD	Incorporated to the sum of measurements	iUPD	Incorporated to the sum of measurements	iUPD	A treatment cycle

Currently, when a patient undergoing ICIs experiences tumor enlargement, although it may be categorized as iUPD instead of PD, the decision to continue treatment often still relies on the subjective judgment of the treating physician. Due to the current lack of objective standards to determine whether tumor growth is genuine or due to pseudo-progression, it is crucial to avoid prematurely discontinuing ICIs for patients exhibiting pseudo-progression. Conversely, for patients with true progression, alternative treatments should be considered. Misjudgment can lead to delays in patient care. Pseudo-progression, as a special form of stable disease, raises questions about whether patients will exhibit very similar tumor growth rate variations, which warrants further investigation in the future (4, 70, 71). Additionally, even genuine disease progression should not be considered solely as an endpoint. For instance, the ratio between the time to progression on early treatment and the time to progression on subsequent treatment (i.e., Growth Modulation Index, GMI), may offer insights into the patient’s long-term prognosis (72). Therefore, future iterations of the RECIST criteria may need to consider incorporating pseudo-progression and hyper-progression into their assessment framework to enhance their accuracy in clinical decision-making. In recent years, the emergence of neoadjuvant therapies has significantly expanded the patient population eligible for surgery. The primary goal of neoadjuvant therapy is to downsize tumors and reduce staging through preoperative chemotherapy combined with ICIs to meet the criteria for surgery. It has been found that RECIST 1.1 criteria can effectively predict disease free survival (dfs) after neoadjuvant therapy for ovarian cancer (73). In the future, it may be necessary to develop more detailed RECIST criteria specifically tailored for patients undergoing neoadjuvant therapy to provide accurate guidance.

RECIST criteria primarily assess the efficacy of tumor treatment by measuring changes in tumor diameter. However, these changes can only be detected when there are sufficient macroscopic alterations in tumor volume. The limitations arise when tumors exhibit early progression or subtle changes, restricting the utility of radiological tools in assessing disease response at early stages. Therefore, further improvements and future directions for RECIST mainly involve two aspects. One is the continuous improvement and innovation of radiological imaging tools. Currently, various imaging tools have emerged, such as Single Photon Emission Computed Tomography (SPECT), molecular Magnetic Resonance Imaging (mMRI), Magnetic Resonance Spectroscopy (MRS), optical imaging (bioluminescence, fluorescence), photoacoustic imaging, and multimodal imaging. Integrating these tools with RECIST criteria poses a significant challenge, as it requires standardizing their application to evaluate lesions and establishing uniform standards. Additionally, exploring new biological imaging biomarkers to serve as novel surrogate endpoints in ICIs regimens should be one of the focal points of research in recent years (74, 75). As a potential direction for the future development of RECIST, it may be beneficial to update the RECIST framework by integrating new technologies or methodologies into its current content. Additionally, when applying RECIST criteria to certain specific diseases, allowances should be made for varying degrees of modification, supported by evidence-based medicine, to adapt to the current clinical landscape (76). Meanwhile, future research should focus on the development of effective treatment strategies based on RECIST criteria, such as various early scoring systems, to address the new challenges brought about by the era of ICIs and targeted therapy, thereby providing more reliable guidance for future clinical practice. It is also important to realize that RECIST may eventually be replaced by a brand-new method or guide, but before that, the constantly revised and developed RECIST standard must be constantly tested in practice and recognized by more consensus. Until then, RECIST 1.1 was still the main body of solid tumor evaluation. Every oncologist in the world remembers that even the progressive diseases seen in the evaluation scan may mean other things, and the treatment will continue as long as the patients have clinical benefits
